# Non-phosgene route: catalytic carbonylation of amines to form unsymmetrical ureas

**DOI:** 10.1093/nsr/nwae450

**Published:** 2024-12-09

**Authors:** Shunji Xie, Ye Wang

**Affiliations:** State Key Laboratory of Physical Chemistry of Solid Surfaces, Collaborative Innovation Center of Chemistry for Energy Materials, Innovation Laboratory for Sciences and Technologies of Energy Materials of Fujian Province (IKKEM), National Engineering Laboratory for Green Chemical Productions of Alcohols, Ethers and Esters, College of Chemistry and Chemical Engineering, Xiamen University, China; State Key Laboratory of Physical Chemistry of Solid Surfaces, Collaborative Innovation Center of Chemistry for Energy Materials, Innovation Laboratory for Sciences and Technologies of Energy Materials of Fujian Province (IKKEM), National Engineering Laboratory for Green Chemical Productions of Alcohols, Ethers and Esters, College of Chemistry and Chemical Engineering, Xiamen University, China

**Figure 1.**
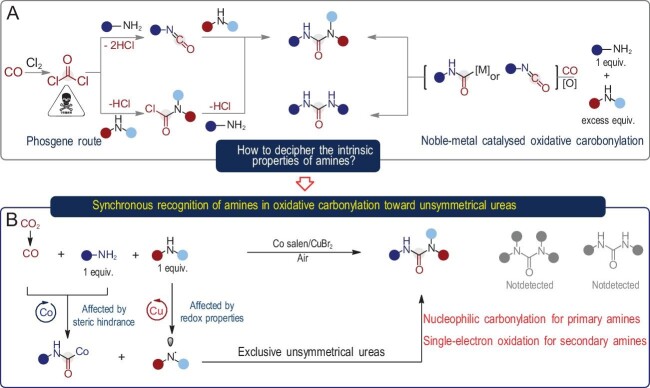
(A) Multi-steps phosgene route for the synthesis of unsymmetrical ureas; transition-metal-catalyzed oxidative carbonylation of amines. (B) Synchronous recognition of amines (nucleophilic carbonylation for primary amines and single-electron oxidation for secondary amines) in oxidative carbonylation toward unsymmetrical ureas.

The production of unsymmetrical ureas, which are widely used in the pharmaceutical field [1], still relies heavily on the multi-steps reaction of two different amines with extremely toxic phosgene as the carbonyl source, resulting in the generation of large amounts of HCl (Fig. 1A) [2]. Therefore, there is a high demand for the development of green alternatives for unsymmetrical urea production. Transition-metal-catalyzed oxidative carbonylation of amines has the potential to be an ideal tool for the synthesis of urea, in which the carbonylation of two amine fragments with CO produces a minimal amount of waste. However, it is very difficult to bind two different amines to a single site, and the formation of symmetrical ureas is unavoidable even when using an excess of one of the two amines (Fig. 1A) [3]. The potential for achieving precise amine recognition stands as a pivotal solution to surmount these obstacles. The realization of this prospect holds the promise of ushering in a transformative era, empowering the ideal oxidative carbonylation of amines in a meticulously controlled 1:1 ratio, thereby facilitating the bespoke synthesis of unsymmetrical ureas.

A recent report in *Science* details a collaborative effort between the Lin He and Aiwen Lei research groups, which has resulted in the direct carbonylative synthesis of unsymmetrical ureas from primary amines/NH_3_ and secondary amines [4]. The key to making this non-phosgene route possible lies in the use of a bimetallic catalytic system that enables the dual process of nucleophilic carbonylation and radical transformation within the confines of a single catalytic cycle. Based on the subtle differences in the physicochemical properties of different amines, a synchronous recognition strategy is proposed: using Co(salen) catalyst for the accurate identification of primary amines/NH_3_ through a sterically controlled nucleophilic carbonylation process to give metal amides, and using Cu salts for the accurate identification of secondary amines through electron effects that preferentially oxidize secondary amines to radical species. As confirmed through the X-ray single-crystal structure, Co(salen)-Br selectively reacts with *^n^*BuNH_2_ to form Co-amide when *^n^*BuNH_2_ and *^n^*Bu_2_NH are added in a 1:1 molar ratio. Conversely, the outcomes of quick-scan X-ray absorption fine structure (XAFS) spectroscopy indicate that Cu^1+^ is produced from CuBr_2_ and *^n^*Bu_2_NH at a considerably faster rate than Cu^1+^ from CuBr_2_ and *^n^*BuNH_2_. With the integrated use of dual amine recognition, complete selectivity for cross-carbonylative coupling is achieved under optimized conditions *via* cobalt/copper bimetallic catalysis, resulting in the exclusive formation of unsymmetrical ureas (Fig. 1B).

Theoretically, CO_2_ should be the ideal carbonyl source in carbonylation reactions. Unfortunately, the high chemical inertness of CO_2_ limits its practical use in carbonylation processes. To enable the conversion of CO_2_ into unsymmetrical urea, the research team then developed an electrothermal catalytic process that utilizes the electrochemical reduction of CO_2_ to CO, followed by copper/cobalt thermocatalytic oxidative carbonylation to produce unsymmetrical ureas with a high chemoselectivity of 93%. Selected examples show that the reactivity of the relay process is comparable to that of the direct CO route (Fig. 1B).

This novel carbonylation process is applicable not only to primary and secondary alkyl amines but also extends the substrate range to encompass various aromatic and halogenated amines. Furthermore, the combination of secondary amines with NH_3_ was demonstrated to be an effective approach to corresponding unsymmetrical ureas. Additionally, intramolecular primary and secondary amines have been observed to undergo intramolecular recognition, resulting in the formation of cyclized unsymmetrical ureas. This method allows for the straightforward synthesis of numerous drug fragments currently on the market, as well as a comprehensive library of examples. In addition to its implications for streamlining pharmaceutical development, this innovation heralds the opening of unexplored horizons in oxidative carbonylation chemistry.
